# Diffusion Tensor Imaging Along the Perivascular Space (DTI-ALPS) in Ischemic Stroke: A Systematic Review of Diagnostic and Prognostic Performance for Post-Stroke Cognitive Impairment

**DOI:** 10.3390/diagnostics15222905

**Published:** 2025-11-17

**Authors:** Mirela Loredana Grigoras, Andrei-Cristian Bondar, Felix Bratosin, Iulia Georgiana Bogdan, Felicia Marc

**Affiliations:** 1 Department of Functional Sciences, Victor Babes University of Medicine and Pharmacy, 300041 Timisoara, Romania; 2 Faculty of General Medicine, “Titu Maiorescu” University, Calea Văcărești 187, 040051 Bucuresti, Romania; 3 Department of Infectious Diseases, Victor Babes University of Medicine and Pharmacy, 300041 Timisoara, Romania; 4 Department of Medical Sciences, Faculty of Medicine and Pharmacy, University of Oradea, 410073 Oradea, Romania

**Keywords:** ischemic stroke, post-stroke cognitive impairment, glymphatic system, perivascular space, diffusion MRI, dementia risk

## Abstract

**Background/Objectives:** Post-stroke cognitive impairment (PSCI) affects ~40% of survivors. Diffusion Tensor Imaging Analysis Along the Perivascular Space (DTI-ALPS) is a fast, contrast-free surrogate of perivascular (glymphatic-aligned) diffusivity that may stratify PSCI risk. We systematically synthesized evidence on the diagnostic and prognostic performance of ALPS in ischemic stroke. **Methods:** Following PRISMA 2020, we searched PubMed/MEDLINE, Scopus, and Web of Science from inception to August 2025 for human ischemic stroke studies reporting ALPS and cognitive or functional outcomes. Eligible designs were cohort or case–control. Outcomes included group differences, associations with cognition (Montreal Cognitive Assessment [MoCA]/Mini-Mental State Examination [MMSE]), prognostic accuracy for PSCI/functional outcome, and longitudinal change. Risk of bias was appraised with QUADAS-2 (diagnostic) and QUIPS (prognostic). The protocol was registered on OSF. Heterogeneity among studies precluded a meta-analysis. **Results:** Five single-center cohorts (*n* per cohort 29–120) from Asia, Europe, and the USA used 3T DTI with ventricular-level ALPS ROIs. Across studies, ALPS was lower after stroke, with early ipsilesional depression and partial recovery over weeks to months. ALPS correlated with cognition (MoCA r ≈ 0.43–0.56) and discriminated early cognitive impairment (AUC 0.868; sensitivity 96%, specificity 66%). Follow-up ALPS predicted poor 6-month outcome (AUC 0.786). In lacunar stroke with small-vessel disease, higher baseline ALPS related to better cognitive trajectories and lower incident dementia risk (HR ≈ 0.33), though associations attenuated after adjustment for diffusion microstructural covariates (PSMD/MD). Reporting of acquisition parameters and ROI methods varied; overall risk of bias was moderate. **Conclusions:** DTI-ALPS shows consistent post-stroke reductions, recovery-sensitive trajectories, and promising—though context-dependent—prognostic value for PSCI and longer-term outcomes. Clinical translation will require standardized acquisition/analysis, multimodal adjustment, prespecified cut-offs, and prospective multicenter validation.

## 1. Introduction

Ischemic stroke remains a leading cause of disability worldwide, and post-stroke cognitive impairment (PSCI) is common, with recent systematic synthesis estimating a pooled prevalence between ~39% and 47% across heterogeneous cohorts and timepoints [1]. Beyond focal tissue loss and diaschisis, converging evidence implicates impaired brain waste–clearance pathways in cognitive decline. The “glymphatic” system—convective cerebrospinal fluid (CSF)–interstitial fluid exchange routed along perivascular spaces (PVS) and supported by perivascularly polarized aquaporin-4 (AQP4)—facilitates removal of metabolites including Aβ and tau [2,3]. Glymphatic transport is modulated by arousal state and circadian biology, with influx and lymphatic efflux peaking during the rest phase and diminishing with loss of AQP4 polarization [4]. In humans with acute ischemic stroke, multimodal work now links glymphatic dysfunction to edema dynamics and functional outcomes, suggesting that perivascular transport failure participates in the cascade from cytotoxic–vasogenic edema to secondary neuronal injury and cognitive sequelae [5]. These mechanistic insights motivate imaging biomarkers that non-invasively index perivascular transport and stratify PSCI risk.

Diffusion Tensor Imaging Analysis Along the Perivascular Space (DTI-ALPS) leverages the orthogonal geometry of periventricular white-matter tracts and medullary vein PVS near the lateral ventricles. By comparing diffusivity aligned with presumed PVS (approximate left–right axis) to diffusivities along projection (superior–inferior) and association (anterior–posterior) fibers, the ALPS index emphasizes motion parallel to PVS; lower values are interpreted as reduced perivascular fluid mobility [6]. ALPS is attractive because it is fast (<5 min), contrast-free, and uses standard DTI; however, rigorous work shows that acquisition parameters, head orientation, and gradient scheme influence absolute values, underscoring the need for standardization and reporting [7]. Multisite harmonization further mitigates scanner/protocol effects and strengthens brain–behavior associations, supporting its use in multi-center studies [8]. Methodological work emphasizes that ALPS is an indirect, regional surrogate for perivascular transport rather than a direct physiological measurement and that it is sensitive to local white-matter geometry, crossing fibers, and free water contamination, all of which can bias interpretation [9,10].

Across neurological disorders, ALPS indices track disease severity and cognition; in stroke specifically, human MRI cohorts consistently demonstrate ipsilateral ALPS reductions early after infarction, partial recovery with time, and associations with cognitive performance. A foundational ischemic stroke study (*n* = 50 vs. 44 controls) reported lower ipsilateral ALPS and a positive association between time-since-onset and ALPS, suggesting dynamic recovery of perivascular transport [11]. A prospective subcortical infarct cohort found lesion-side ALPS correlated with MoCA at 7 and 90 days and discriminated early cognitive impairment with an AUC of 0.868 [12]. In a separate cohort, the infarct-side ALPS at follow-up predicted poor outcomes at 6 months (AUC 0.786), and was associated with PSCI at 6 months, complementing links to NIHSS and MMSE [13]. Beyond the subacute window, in lacunar stroke with confluent white-matter hyperintensities, higher baseline ALPS predicted lower incident dementia risk over five years (HR ≈ 0.33), even after adjustment for conventional diffusion metrics [14]. Together, these data position ALPS as a promising candidate biomarker for PSCI risk stratification and monitoring.

Despite multiple single-center cohorts and burgeoning interest, we found no PRISMA-compliant, stroke-specific systematic review consolidating ALPS effect sizes, ipsilateral–contralateral asymmetries, time-courses, and prognostic accuracy for PSCI. Existing reviews either survey glymphatic imaging across disparate diseases or discuss ALPS methods and theory without systematically aggregating stroke outcomes; where stroke is covered, it is typically narrative and not limited to ischemic etiologies [9,15]. As ALPS enters early clinical research use, trialists and clinicians require a consolidated view of its diagnostic and prognostic performance specifically in ischemic stroke—along with sources of heterogeneity (timing post-stroke, lesion topography, small-vessel disease burden), and analytic pitfalls (ROI placement, crossing fibers, free water contamination). Our review addresses this gap with an emphasis on PSCI-relevant endpoints (MoCA/MMSE, 6-month outcomes, and long-term dementia conversion), methodological quality, and interpretability.

An ideal PSCI biomarker should be non-invasive, rapid, repeatable, and biologically interpretable. ALPS can be integrated into standard diffusion acquisitions within minutes, enabling serial assessments across acute, subacute, and chronic phases. Standardization and harmonization work suggests that with fixed planes/orientation, consistent gradient schemes, and cross-site harmonization, ALPS achieves acceptable reproducibility—facilitating longitudinal evaluation of perivascular “recovery phenotypes” and potential treatment response [7,8]. If ALPS adds information beyond lesion size and white-matter hyperintensity burden—by indexing edema clearance and perivascular function—it could help identify patients for targeted cognitive rehabilitation, sleep/circadian optimization, or future trials of glymphatic-modulating interventions. In routine practice, ALPS may complement existing structural markers, providing mechanistic context for PSCI risk and a non-contrast endpoint for follow-up.

Accordingly, we systematically collate human ischemic stroke studies that (i) compare ALPS between stroke and controls and between ipsilateral and contralateral hemispheres; (ii) test associations with MoCA/MMSE across acute–subacute windows; (iii) evaluate prognostic utility for 6-month outcomes and incident dementia in longer-term cohorts; and (iv) report longitudinal ALPS trajectories during recovery. We also summarize acquisition/analysis features, discuss interpretability in light of crossing-fiber and free water confounds, and map knowledge gaps to a research agenda aimed at standardization and clinical translation.

## 2. Materials and Methods

### 2.1. Protocol and Registration

Following PRISMA 2020 [16], we searched PubMed/MEDLINE, Scopus, and Web of Science from inception to August 2025 for human ischemic stroke studies reporting ALPS. The review question asked whether, in adults with ischemic stroke (including lacunar infarction), the DTI-ALPS index differs from appropriate comparators and whether it associates with or predicts post-stroke cognitive outcomes. PICO: Population—adults with ischemic stroke; transient ischemic attack (TIA) was not part of the target population and was excluded at eligibility unless ischemic stroke–specific results were separable; Index—DTI-ALPS; Comparator—healthy or non-stroke controls and/or the contralateral hemisphere; Outcomes—ALPS group differences, associations with MoCA/MMSE or PSCI, prognostic discrimination, and longitudinal change. Accordingly, only cohorts with ischemic stroke and separable ischemic stroke–specific outcome reporting were retained for analysis; mixed TIA/small-vessel disease samples without distinct ischemic stroke data were excluded. Eligible designs were prospective or retrospective cohort or case–control human studies published as full-text, peer-reviewed articles.

The protocol was registered on OSF (osf.io/a8ght). Deviations: (i) one broadened search rerun (24 August 2025) to ensure capture of 2024–2025 studies; (ii) adoption of a SWiM narrative synthesis due to heterogeneity precluding meta-analysis; (iii) pre-specification that, when 95% CIs were not reported, we would compute binomial exact CIs for sensitivity/specificity from reported counts when available and otherwise flag as unavailable.

### 2.2. Eligibility Criteria

Eligibility criteria admitted original human studies in adults with ischemic stroke that reported DTI-ALPS values with extractable numeric data and at least one clinical, cognitive, functional, or longitudinal imaging outcome relevant to PSCI or recovery. Mixed cohorts were eligible only when ischemic stroke–specific results were extractable; TIA-only cohorts were excluded as out-of-scope for the defined population. Exclusions comprised single-case reports without numeric ALPS values, pediatric-only cohorts, non-ischemic etiologies alone (such as isolated intracerebral hemorrhage) without ischemic data, conference abstracts lacking sufficient numbers, narrative reviews, methodological notes without patient data, and animal-only research. When cohort overlap was suspected, the most complete and least redundant dataset was retained. An outcomes hierarchy prioritized MoCA over MMSE for early PSCI when both were available; validated PSCI case definitions were abstracted when provided. For ALPS, lesion-side values were prioritized for association analyses, while hemispheric asymmetry and bilateral/global ALPS were abstracted when reported. Unit-of-analysis issues (per hemisphere vs. per subject) were recorded, with preference for per-subject summaries when available.

### 2.3. Information Sources and Search Strategies

Three electronic databases (PubMed/MEDLINE, Scopus, and Web of Science Core Collection) were searched from inception to 24 August 2025. Search strategies combined (i) construct-specific terms related to DTI-ALPS, perivascular spaces, and glymphatic function, and (ii) cerebrovascular disease terms (ischemic stroke, lacunar infarction, small-vessel disease, transient ischemic attack), with database-specific limits to human, English-language, peer-reviewed original articles. A summary of search fields, key limits, and representative term blocks for each database is provided in Table 1. The complete Boolean search strings, including all operators and limits for each database, are provided in Supplementary Table A1 for reproducibility. Transient ischemic attack and small-vessel disease terms were intentionally included to maximize recall; however, studies limited to TIA or mixed cohorts without separable ischemic stroke data were excluded at full-text screening, as detailed in the eligibility criteria. Forward and backward citation chasing was additionally performed on all included records to identify any missed eligible studies.

### 2.4. Selection Process

**Figure 1:**
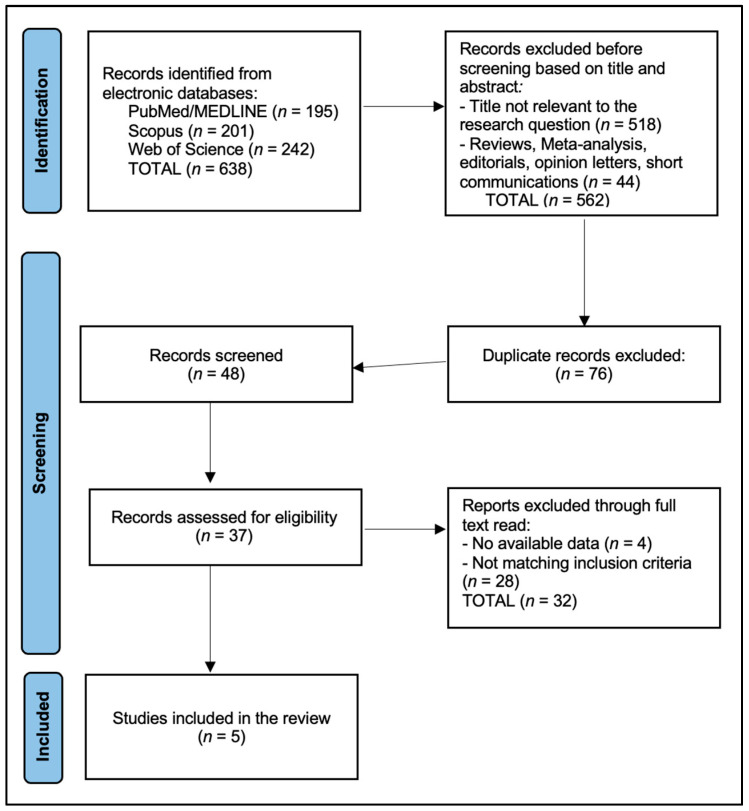
PRISMA Flowchart Diagram. Records identified *n* = 638; duplicates removed *n* = 76; screened *n* = 48; title/abstract excluded *n* = 11; full-text assessed *n* = 37; at full-text screening, 32 reports were excluded. The main reasons were: not ischemic stroke–specific ALPS (*n* = 12), wrong outcomes (no relevant clinical/cognitive/longitudinal endpoints; *n* = 8), insufficient extractable quantitative ALPS data (*n* = 4), cohort or analysis overlap with a more complete dataset (*n* = 4), and non-human or pediatric-only populations (*n* = 4).

Study selection proceeded in duplicate at the title/abstract level followed by full-text assessment against eligibility criteria, with discrepancies resolved by consensus and reasons for exclusion recorded (population, index test not ALPS, no extractable numbers, non-ischemic only, wrong outcomes, overlap). Data extraction captured study design, country/setting, sample sizes (stroke/control), stroke subtype(s) and timing post-event (acute, subacute, chronic), DTI acquisition parameters when stated (field strength, b-values, number of directions, motion/eddy handling), ALPS implementation details (ROI placement method, hemispheric specification, formula, any automation), and all prespecified outcomes: ALPS differences (stroke vs. control; lesion vs. non-lesion), correlations with cognition (MoCA/MMSE), discrimination for PSCI or functional outcomes (AUC with 95% CI, sensitivity/specificity, cut-offs), longitudinal change, and relationships with edema or small-vessel disease markers (WMH, PSMD, MD, PVS volume), as well as incident dementia or long-term cognitive change when available. Covariates, modeling strategies, handling of missing data, and numerical reporting format (tables vs. figure-only) were noted. When only graphical results were presented, extraction relied on numeric labels if provided; otherwise, values were marked as not reported (NR). Reasons for exclusion at the full-text stage were recorded, and the study flow is summarized in the PRISMA diagram (Figure 1). Predefined reasons included “TIA-only or not ischemic stroke–specific” for population misalignment.

We identified 638 records (PubMed/MEDLINE 195; Scopus 201; Web of Science Core Collection 242). After cross-source deduplication (76 duplicates removed), 48 unique records were screened at title/abstract; 37 full texts were assessed; and 5 studies were included (Figure 1). A source-wise pre/post-deduplication table is provided below in Table 2.

For discrimination, we extracted AUCs (with 95% CIs and thresholds), sensitivity/specificity (with 95% CIs), and model covariates. When authors did not report CIs but provided numerators/denominators, we computed exact binomial CIs for sensitivity/specificity and Wald CIs for mean-based contrasts. If neither CIs nor the necessary counts were available, we flagged the cell as “not available” and noted this in the risk-of-bias narrative.

### 2.5. Risk of Bias

Risk of bias was appraised using a domain-based approach aligned to QUADAS-2 for diagnostic/discriminative analyses and QUIPS for prognostic associations, considering participant selection; index-test measurement quality (ALPS acquisition details, ROI placement blinding or automation, reproducibility, scanner variability); outcome assessment (validity of cognitive tests, PSCI definitions, blinding of assessors); confounding (age, sex, education, WMH burden, PSMD/MD, lesion characteristics); attrition; and selective reporting. Studies that adjusted for WMH and peak width of skeletonized mean diffusivity (PSMD) and/or mean diffusivity (MD) were judged at lower concern for confounding in the index-test domain. Particular attention was paid to construct validity given the surrogate nature of ALPS for perivascular transport and the potential influence of fiber architecture, free water, and white-matter disease. Two reviewers independently rated risk of bias (QUADAS-2 for diagnostic/discriminative analyses; QUIPS for prognostic analyses), resolving disagreements by consensus.

### 2.6. Synthesis Approach and Meta-Analysis Decision Rule

We used a SWiM approach with pre-specified grouping by (i) timing: acute (≤14 days), subacute (15–90 days), and chronic (≥3 months). For descriptive purposes, we refer to 3–6 months as an ‘early-chronic’ subset within the chronic window where this helps interpret trajectories; (ii) ALPS implementation: lesion-side vs. contralateral (hemispheric asymmetry) versus global ALPS; and (iii) small-vessel disease burden (presence of confluent WMH or lacunar SVD cohorts). Within groups, we prioritized adjusted analyses over unadjusted. Direction-of-effect vote-counting was weighted by risk-of-bias domain severity (QUADAS-2/QUIPS) and extractability of statistics.

Quantitative pooling was not performed because fewer than three independent studies reported harmonized estimates within any clinically homogeneous subgroup and because of key incompatibilities in imaging timing, ALPS implementation, scanner/DTI protocols, and outcome constructs. Imaging timepoints ranged from 7 days to 5 years post-stroke; ALPS indices were derived from lesion-side vs. contralateral hemispheres or global automated ROIs; scanner vendors, b-values, and diffusion directions differed; and cognitive endpoints spanned MoCA, MMSE, multidomain composites, and adjudicated incident dementia. In this context, a Synthesis Without Meta-analysis (SWiM) approach with direction-of-effect vote-counting was considered more appropriate than a formal meta-analysis.

Where authors reported group means and standard deviations, we computed 95% confidence intervals using a normal approximation (mean ± 1.96 × SD/√*n*), and this computation is noted in the table footnote. We did not compute or impute 95% CIs for AUCs, hazard ratios, or other multivariable estimates when numerators/denominators or standard errors were not available; such entries are explicitly labeled as ‘95% CI not reported’ or ‘NR’. For ALPS values, we abstracted per-subject indices whenever authors provided subject-level summaries; when only hemisphere-wise values (lesion vs. contralateral) were reported, we treated hemispheres as paired within-subject measures and reported them descriptively without cross-study pooling.

## 3. Results

Across five single-center cohorts spanning Taiwan, China, the UK, and a U. S dataset re-analyzed in China, designs ranged from retrospective stroke series to prospective subcortical or lacunar stroke cohorts, with imaging primarily at 3 T using conventional DTI and ALPS ROIs placed at the lateral ventricle level. Toh (*n* = 50) obtained scans within 60 days post-onset and used manual ROIs [17]; Wang (*n* = 29) acquired DTI at 7 and 90 days following acute subcortical infarcts with protocolized ROIs [18]; Zhang (*n* = 51) sampled two timepoints around rehabilitation and included healthy controls and a “non-VCI” comparator group with side-specific ALPS [19]. Hong’s larger lacunar stroke cohort (*n* = 120) implemented a standardized diffusion pipeline over three MRI years with cognitive follow-up to five years [20], while Chen (*n* = 51 stroke; *n* = 27 controls) evaluated chronic-phase trajectories at 3 and 12 months using a multi-direction DTI sequence and lesion/contralateral ROIs [21], as described in Table 3.

ALPS indices were consistently depressed after ischemic stroke, most prominently on the lesion side early. In Toh, the lesion-side mean was 1.162 ± 0.126 versus 1.335 ± 0.160 contralaterally (*p* < 0.001), with values increasing with time since onset [17]. In Wang, both lesion (1.371 ± 0.170) and non-lesion sides (1.310 ± 0.198) were lower than controls (1.568 ± 0.115; both *p* < 0.001), while the within-patient lesion–non-lesion difference was not significant [18]. Zhang reported lower bilateral ALPS acutely with improvement at a short follow-up (exact means not reported) [19]; Hong observed longitudinal declines in global ALPS over three years in lacunar stroke [20]; and Chen found lesion < contralateral at 3 months with hemispheric symmetry largely restored by 12 months [21], as presented in Table 4 and Table 5.

**Figure 2:**
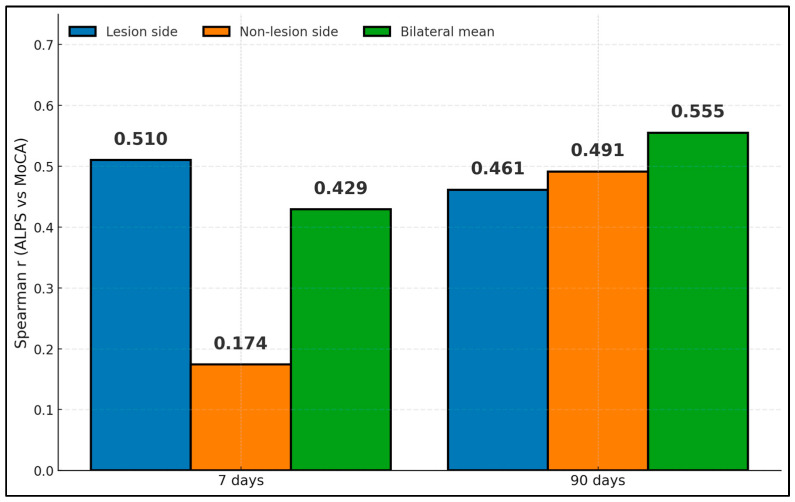
ALPS–MoCA correlations by hemisphere and time.

Spearman correlations from Wang reveal moderate associations between ALPS and MoCA that strengthen with bilateral averaging and at 90 days [18]. At 7 days, r was 0.510 (lesion), 0.174 (non-lesion), and 0.429 (bilateral mean); at 90 days, r was 0.461 (lesion), 0.491 (non-lesion), and 0.555 (bilateral mean). These findings suggest that glymphatic-aligned diffusivity relates to cognitive performance early after subcortical infarct, with bilateral or later assessments capturing the relationship more robustly (Figure 2).

Across studies reporting diagnostic/prognostic performance, effects are presented with 95% CIs when reported by the original authors or otherwise labeled as ‘95% CI not reported’ (Table 6). For Wang (2025) [18], the AUC for early cognitive impairment was 0.868, with sensitivity at 96% and specificity at 66%, but 95% CIs and 2 × 2 counts were not reported and were therefore not reconstructed. Long-term incident dementia in Hong (2024) [20] showed an HR of 0.328 per higher baseline ALPS, with 95% CIs not provided in the main text; we retained this estimate without imputing CIs and flagged the missing uncertainty in our risk-of-bias narrative.

Demographics skewed toward late-middle-aged to older adults (mean 53–70 years) with male predominance. Imaging schedules captured acute/subacute (1–60 days in Toh [17]; 7 and 90 days in Wang [18]) and early-chronic/chronic phases (3 months to 1 year in Chen [21]; annual MRIs over 3 years in Hong [20]), while Zhang sampled pre- and post-rehabilitation intervals with an onset-to-scan threshold around 42 days [19]. DTI parameters were typical (b = 1000 s/mm^2^; 20–32 directions; 2 mm voxels), and cognitive endpoints varied: MoCA at 7/90 days [18], MMSE and PSCI at 6 months [19], and multi-domain batteries or incident dementia over long-term follow-up [20,21], as presented in Table 7.

**Figure 3:**
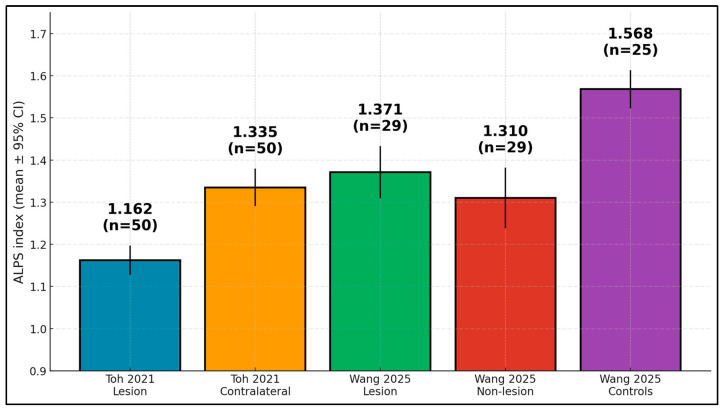
ALPS index by group—lower in stroke hemispheres than controls (means ± 95% CI; *n* shown).

The findings show lower ALPS in stroke hemispheres versus controls, with values annotated by sample sizes. In Toh, lesion-side ALPS was 1.162 (*n* = 50) and contralateral 1.335 [17]; in Wang, lesion and non-lesion values were 1.371 and 1.310 (both *n* = 29), while controls measured 1.568 (*n* = 25) [18], as presented in Figure 3.

Methodological heterogeneity spanned participant selection (retrospective vs. prospective), imaging timing (acute/subacute vs. chronic), DTI parameters (b-value, number of directions, voxel size), the ALPS ROI method (manual vs. standardized/automated, side-specific vs. global), cognitive endpoints (MoCA vs. MMSE vs. multidomain/incident dementia), and covariate adjustment (PSMD/MD, WMH). These differences are summarized in Table 8 and collectively motivated a SWiM (synthesis without meta-analysis) approach rather than formal pooling.

We produced a domain-level risk-of-bias assessment and created two tables: Table 9 (QUADAS-2) for diagnostic/discriminative analyses and Table 10 (QUIPS) for prognostic analyses, with brief domain-specific rationales. Common concerns were: (i) participant selection in retrospective designs (Toh [17], Chen [21]), (ii) index-test measurement where ROIs were manual or blinding was unclear (Toh [17]; unclear in Wang/Zhang [18,19]), and (iii) residual confounding where PSMD/MD and WMH were not modeled (Wang, Zhang, Chen [18,19,21]). The long-term lacunar cohort (Hong) showed lower concerns due to standardized pipelines and diffusion covariate adjustment.

## 4. Discussion

### 4.1. Summary of Evidence

Across five studies, DTI-ALPS is lower after ischemic stroke, often more on the infarct side early, and relates to short-term cognition, 6-month outcomes, and long-term dementia risk in SVD. Longitudinal data support partial ALPS recovery, with prognostic value more apparent at follow-up than at baseline in some cohorts (Time-2 ALPS predicting poor outcome at 6 months). These findings collectively position ALPS as an emerging, rapid, non-contrast MRI research marker that may complement conventional stroke imaging, while any diagnostic or prognostic role in clinical practice will require standardized acquisition and external validation.

Translational work suggests that glymphatic disruption is associated with edema dynamics and outcomes, and human stroke cohorts show compatible trends. Similarly, one study demonstrated that edema evolution aligned better with glymphatic impairment than with BBB closure timing; pharmacologic enhancement of glymphatic function mitigated edema and improved cognition in models [22]. Clinically, lower ALPS may reflect impaired perivascular drainage and free water shifts that contribute to neuroinflammation and PSCI.

Important critiques emphasize that ALPS is an indirect, regional surrogate that can be confounded by fiber geometry, WMH, age, and scanner differences. Bayesian/meta-analyses and methodological reviews call for harmonized pipelines and for ALPS to be interpreted alongside conventional diffusion metrics (MD, PSMD) and vascular lesion burdens. The attenuation of ALPS change after PSMD/MD adjustment in the lacunar cohort illustrates this issue and argues for multimodal models [23].

Underpinning cognitive associations in ischemic stroke, independent cohorts show that ALPS co-varies with motor outcomes, consistent with interpretation of ALPS as a recovery-sensitive marker rather than a cognition-specific correlate, although mechanistic specificity remains limited. In subacute unilateral subcortical infarcts, ipsilesional ALPS was reduced versus the non-lesioned hemisphere and related to Fugl–Meyer motor scores (ρ ≈ 0.52), with complementary links to corticospinal microstructure—patterns consistent with hemispheric asymmetries and partial normalization observed across stroke cohorts. [24].

Hemodynamic manipulation has been observed to modulate ALPS in some cohorts, in a manner consistent with the ‘pulsatility–neurofluid’ hypothesis, although these observations remain associative. In severe carotid stenosis, ALPS increased within 24 h of angioplasty/stenting and the magnitude of early change correlated with two-month MMSE gains (rs ≈ 0.47). High inter-reader agreement was reported. These peri-procedural dynamics align with trajectories described in stroke cohorts and suggest that ALPS can register upstream improvements in arterial pulsatility and neurofluid coupling—features desirable in a treatment-responsive biomarker for PSCI [25].

Longitudinal small-vessel disease data strengthen the case for composite modeling. Over ~3.4 years, lower baseline ALPS and higher free water independently tracked greater WMH/lacune progression and slower processing speed; free water also predicted MMSE decline, while ALPS related to change in global cognition and processing speed. Mediation analyses in CSVD further indicate that ALPS partly transmits the effect of WMH burden on episodic memory. Taken together, these results argue for joint models (ALPS + free water + WMH/PSMD) when estimating PSCI risk rather than treating ALPS as a stand-alone proxy for perivascular transport [26,27].

Population-based imaging offers construct-validity cautions that complement methodological recommendations. In a 2715-participant community cohort, automated ALPS was robust across scanners but associated primarily with age, vascular risk, and WMH, and only minimally with amyloid/tau; authors concluded that ALPS may better index vascular/white-matter processes than Alzheimer-specific biology. Similarly, analysis of 17,723 UK Biobank scans replicated associations with age, sleep continuity, cognition, and sex, but with small effect sizes, underscoring the need for standardized acquisition and careful covariate handling when applying ALPS to PSCI stratification [28,29].

Cross-disease evidence positions ALPS on a broader vulnerability axis for cognitive decline. In Parkinson’s disease with MCI, lower baseline ALPS identified converters to dementia (AUC ≈ 0.85) and related to basal ganglia PVS burden and executive/global performance. Although pathophysiology differs from ischemic stroke, these findings reinforce the prognostic potential of ALPS for cognitive outcomes and motivate adjudicated endpoints and harmonized cut-points in PSCI research [30].

Population-scale resources can anchor clinical interpretation and study design. UK Biobank–derived distributions enable practical z-scoring by age/sex, while a genome- and phenome-wide study of ~40,000 individuals linked lower ALPS to stroke risk (with obesity as a mediator) and identified detailed genetic determinants—context that informs prevention-oriented PSCI programs. For translation, a prespecified pipeline that pairs serial ALPS with free water/WMH, standardizes DTI planes and gradients, employs automated ROIs with QC, and samples acute (≤2 weeks), subacute (6–8 weeks), and 6–12-month timepoints is likely to yield more reproducible risk estimates and clearer mechanistic inference [29,31].

While reduced ALPS is often interpreted as impaired glymphatic-aligned transport, several co-existing processes likely contribute to ALPS dynamics after ischemic stroke. First, cytotoxic/vasogenic edema elevates interstitial pressure and shifts free water, plausibly damping perivascular-aligned diffusivity and temporally tracking outcomes [22]. Second, neuroinflammation may disrupt PVS structure and aquaporin polarity, degrading perivascular conduit function. Third, cerebral hypoperfusion reduces arterial pulsatility—the presumed driver of perivascular exchange—consistent with observations that ALPS increases within 24 h after carotid revascularization and tracks short-term cognitive gains [25]. Finally, diffusion microstructural injury and WMH burden can confound ALPS associations, as illustrated by attenuation after PSMD/MD adjustment in small-vessel cohorts [20,26]. Accordingly, ALPS should be interpreted within multimodal models (ALPS + free water + WMH/PSMD) rather than as a stand-alone proxy for “perivascular transport”.

Beyond focal infarction, converging evidence underscores the contribution of diffuse small-vessel disease and microstructural disintegration to PSCI. Cerebral small-vessel disease imaging markers—white-matter hyperintensities, lacunes, and peak width of skeletonized mean diffusivity (PSMD)—show robust associations with post-stroke cognitive decline and incident dementia, and in some cohorts ALPS and PSMD appear to capture overlapping aspects of vascular microstructural injury [20,26,27]. In Hong’s lacunar stroke cohort, for example, baseline ALPS related to long-term cognitive trajectories and incident dementia, but its effect attenuated once PSMD and mean diffusivity were added to the models [20]. These data support a framework in which ALPS is interpreted alongside established vascular injury markers rather than in isolation, and suggest that integrating ALPS with PSMD, WMH burden, and free water indices may yield more stable and biologically grounded risk estimates for PSCI.

Near-term clinical use cases include screening for early PSCI risk (favoring sensitivity), triaging for cognitive rehab, and serving as a mechanistic endpoint in interventions targeting edema/perivascular function. Standardization priorities: (i) prespecified DTI b-values/directions; (ii) automated ROI (atlas-based) with QC; (iii) harmonized cognitive batteries and PSCI definitions; (iv) ALPS + WMH/free water joint modeling; and (v) multi-timepoint designs (acute, 6–8 weeks, 6–12 months). Pragmatically, adding a 4–5 min DTI-ALPS slice to standard stroke MRI is feasible and may enhance risk stratification pending validation.

### 4.2. Limitations

The evidence base is small and heterogeneous; most cohorts are single-center studies of modest sample size; DTI protocols and ROI placement vary; and several studies report incomplete acquisition details (NR cells). Critically, ALPS is not a direct perivascular transport measurement and overlaps with microstructural diffusion signals; the Hong 2024 attenuation after PSMD/MD adjustment underscores this [20]. We synthesized narratively without meta-analysis due to design/outcome heterogeneity. Moreover, publication bias is possible. We excluded TIA unless ischemic-specific results were separable, improving construct specificity but potentially limiting generalizability to very mild ischemic presentations that clinically resemble TIA. Across diagnostic/discriminative questions, the domains with higher concern were related to index-test measurement (manual ROIs; unclear blinding) and flow/timing (broad windows), which inflate between-study variance and may overstate early hemispheric asymmetry. Prognostic inferences were most sensitive to confounding (absence of PSMD/MD/WMH adjustment in Wang/Zhang/Chen [18,19,21]) and statistical reporting (lack of CIs or model details). Where PSMD/MD were modeled (Hong [20]), ALPS associations attenuated, suggesting that perivascular-aligned diffusivity is partly collinear with microstructural injury. Accordingly, we weight conclusions towards (i) consistent lesion-side depression and recovery sensitivity (lower RoB), and (ii) context-dependent prognostic value that is strongest when ALPS is considered with microstructural covariates, rather than alone. Future work should adjudicate minor ischemic events with standardized imaging and cognitive follow-up.

## 5. Conclusions

DTI-ALPS shows consistent post-stroke reductions, tracks recovery, and associates with cognition and outcomes, including incident dementia in small-vessel disease, supporting its potential as a non-contrast, rapid imaging marker for PSCI risk and mechanistic monitoring in research settings. However, clinical adoption awaits standardized acquisition/analysis, multimodal adjustment (WMH, free water), and prospective multicenter validation with prespecified cutoffs and patient-centered endpoints.

## Figures and Tables

**Table 1:** Databases searched and key search strategy characteristics.

Database/Platform	Search Field(s) and Coverage	Limits and Filters	Core Search Concepts and Representative Terms *
PubMed/MEDLINE	Title/Abstract ([tiab]) with database filters; coverage from 1900 to 24 August 2025	Humans; English; Article-type; exclusion of reviews; date range 1900–2025; animals excluded except “humans”	DTI-ALPS/glymphatic/PVS block: “DTI-ALPS”, “ALPS index”, “analysis along the perivascular space”, “perivascular space(s)”, “Virchow-Robin” combined with diffus */tensor/DTI/“diffusion tensor imaging”/diffusivity/“fractional anisotropy”/“mean diffusivity”; “glymphatic”/“glymphatic system” combined with diffus */DTI. Stroke/cerebrovascular block: stroke, ischem *, ischaem *, infarct *, lacunar, “lacunar stroke”, “small vessel disease”, “small-vessel disease”, SVD, “transient ischemic attack”, TIA, lacune *, “subcortical infarct”, “subcortical infarcts”.
Scopus (Elsevier)	TITLE-ABS-KEY; coverage from 1900 to 24 August 2025	Document type: Article; Language: English; Publication years >1900 and <2026	Same concept blocks as PubMed, adapted to Scopus syntax: TITLE-ABS-KEY(“DTI-ALPS” OR “ALPS index” OR “analysis along the perivascular space” OR ((perivascular OR “perivascular space” OR “perivascular spaces” OR “Virchow-Robin”) AND (diffus * OR tensor OR DTI OR “diffusion tensor imaging” OR diffusivity OR “fractional anisotropy” OR “mean diffusivity”)) OR ((glymphatic OR “glymphatic system”) AND (diffus * OR DTI OR “diffusion tensor imaging”))) AND TITLE-ABS-KEY (stroke OR ischem * OR ischaem * OR infarct * OR lacunar OR “lacunar stroke” OR “small vessel disease” OR “small-vessel disease” OR SVD OR “transient ischemic attack” OR TIA OR lacune * OR “subcortical infarct” OR “subcortical infarcts”).
Web of Science Core Collection	Topic field (TS); coverage from 1900 to 24 August 2025	Document type: Article; Language: English; Publication years 1900–2025	TS = ((“DTI-ALPS” OR “DTI ALPS” OR “ALPS index” OR “ALPS-index” OR “analysis along the perivascular space” OR ((perivascular OR “perivascular space” OR “perivascular spaces” OR “Virchow-Robin”) NEAR/3 (diffus * OR tensor OR DTI OR “diffusion tensor imaging” OR diffusivity OR “fractional anisotropy” OR “mean diffusivity”)) OR ((glymphatic OR “glymphatic system”) NEAR/3 (diffus * OR DTI OR “diffusion tensor imaging”))) AND (stroke OR ischem * OR ischaem * OR infarct * OR lacunar OR “lacunar stroke” OR “small vessel disease” OR “small-vessel disease” OR SVD OR “transient ischemic attack” OR TIA OR lacune * OR “subcortical infarct” OR “subcortical infarcts”)).

* TIA and small-vessel disease terms were included to maximize recall; TIA-only cohorts or mixed cohorts without separable ischemic stroke results were excluded at full-text screening.

**Table 2:** Source-wise search accounting and deduplication.

Source	Records Retrieved (Initial + Rerun)	After Intra-Source Dedupe	Unique After Cross-Source Merge *
PubMed/MEDLINE	195	195	22
Scopus	201	201	13
Web of Science Core Collection	242	242	13
Total	638	638	48

* Primary record assigned to PubMed when available, else Scopus, else Web of Science.

**Table 3:** Study characteristics and imaging methods (DTI and ALPS implementation).

First Author (Year)	Country/Setting	Design and Stroke Cohort	Imaging Timing vs. Stroke (Window)	Controls (*n*)	Scanner and DTI Parameters	ALPS ROI Method/Atlas	PSMD/WMH Covariates in ALPS Models
Toh (2021) [17]	Taiwan (single-center)	Retrospective; *n* = 50 ischemic stroke	1–60 days post-onset (acute/subacute)	44	3 T Siemens Tim Trio; b = 1000 s/mm^2^; 20 diffusion directions; 2 mm slices	Manual periventricular ROIs at lateral ventricle level; no atlas-based automation reported	PSMD and WMH not included; other covariates NR
Wang (2025) [18]	China (single-center)	Prospective acute subcortical infarcts; *n* = 29	7 and 90 days (acute and subacute)	25	3 T Philips Ingenia; b = 1000 s/mm^2^; 32 diffusion directions; 2 mm isotropic voxels	Protocolized lesion and non-lesion ROIs following ALPS method; atlas use NR	Models did not include PSMD/WMH; limited clinical covariates reported; diffusion covariates NR
Zhang (2025) [19]	China (single-center)	Prospective; *n* = 51 ischemic stroke (plus HC and non-VCI comparators)	Time-1 pre-rehab (mostly subacute ≤ 90 days); Time-2 ≈ 30 days later (subacute/early-chronic)	48 HC; 47 non-VCI	3 T GE MR750; DTI parameters reported in supplement (b-value and directions NR in main text)	Side-specific ALPS with additional PVS/choroid-plexus metrics; ROI framework described; atlas type NR	Imaging covariates partially modeled; PSMD/WMH covariates NR
Hong (2024) [20]	UK (Cambridge)	Prospective lacunar SVD cohort; *n* = 120	Baseline; annual MRI over 3 years; cognition over 5 years (chronic)	—	DTI acquired on 3 T scanners using a standardized pipeline; detailed b-values/directions NR in main text	Global ALPS derived from automated/standardized pipeline; ROIs based on white-matter skeleton and periventricular regions; specific atlas NR	PSMD and MD included as covariates; WMH burden incorporated in SVD characterization
Chen (2025) [21]	USA dataset; re-analysis in China	Retrospective chronic stroke; *n* = 51; HC *n* = 27	3 months and 12 months post-stroke (chronic)	27	3 T Siemens Tim-Trio; multi-direction DTI; exact b-value and number of directions NR	Lesion-side and contralateral ROIs using an ALPS-type framework; atlas use NR	PSMD/WMH covariates not modeled; other covariates limited; details NR

ALPS—analysis along the perivascular space; DTI—diffusion tensor imaging; ROI—region of interest; WMH—white-matter hyperintensities; PSMD—peak width of skeletonized mean diffusivity; MD—mean diffusivity; HC—healthy controls; VCI—vascular cognitive impairment; NR—Not Reported.

**Table 4:** ALPS values with uncertainty for studies with extractable means and SD.

Study	Group	Mean (SD)	*n*	95% CI (Computed)
Toh (2021) [17]	Lesion hemisphere	1.162 (0.126)	50	1.127–1.197
Toh (2021) [17]	Contralateral	1.335 (0.160)	50	1.291–1.379
Wang (2025) [18]	Lesion hemisphere	1.371 (0.170)	29	1.309–1.433
Wang (2025) [18]	Non-lesion	1.310 (0.198)	29	1.238–1.382
Wang (2025) [18]	Controls	1.568 (0.115)	25	1.523–1.613

ALPS—analysis along the perivascular space; 95% CIs computed as mean ± 1.96 × SD/√*n* using the reported SDs and sample sizes (*n*). All ALPS values in this table are hemisphere-level indices; *n* corresponds to the number of participants, and lesion vs. contralateral hemispheres are paired within subjects.

**Table 5:** Direction-of-effect summary.

Study	Stroke vs. Controls	Lesion vs. Contralateral	Longitudinal Change
Zhang (2025) [19]	↓ (stroke < controls)	↓ at Time-1; partial ↑ at Time-2	↑ (partial recovery)
Hong (2024) [20]	NR	NR	↓ (decline over 3 years)
Chen (2025) [21]	↓ vs. controls (3 m and 12 m)	Lesion < contralateral at 3 months; ≈by 12 months	≈symmetry by 12 months

y—years; m—meters.

**Table 6:** Cognitive and prognostic associations with standardized reporting.

Study	Cognitive Endpoints	Association Metrics	Prognostic Metrics	Other Outcomes
Toh (2021) [17]	NR	ALPS ↑ with time since onset (β ≈ 0.79, *p* < 0.001)	NR	Hemispheric ALPS asymmetry early post-stroke
Wang (2025) [18]	MoCA at 7 and 90 days	Lesion-side ALPS–MoCA r = 0.510 (7 days), r = 0.461 (90 days); mean bilateral r = 0.429 (7 days), 0.555 (90 days)	AUC 0.868 for early cognitive impairment (sensitivity 96%, specificity 66%)	NR
Zhang (2025) [19]	MMSE; PSCI at 6 months	Time-1 lesion ALPS associated with 6-month MMSE; bilateral WM-PVS also associated	Time-2 lesion ALPS AUC 0.786 for poor 6-month outcome (cutoff ~1.105)	Infarct-side ALPS improved over time
Hong (2024) [20]	Global, memory, executive, processing speed (5 years)	Baseline ALPS associated with change in global cognition and executive and long-term memory (β 0.142–0.287)	Incident dementia HR 0.328 per higher baseline ALPS; change in ALPS predicted dementia univariately but attenuated after PSMD/MD	PVS volume did not predict dementia
Chen (2025) [21]	Multidomain battery at 3 months and 1 year	Weak domain correlations at 3 months (uncorrected), not persisting at 1 year	NR	Hemispheric ALPS gap resolves by 1 year

MoCA—Montreal Cognitive Assessment; MMSE—Mini-Mental State Examination; AUC—area under the receiver-operating characteristic curve; HR—hazard ratio; PSMD—peak width of skeletonized mean diffusivity; MD—mean diffusivity; PSCI—post-stroke cognitive impairment; Wang (2025) [18]: AUC 0.868 (95% CI not reported), sensitivity 96%, specificity 66% (95% CIs not reconstructable without 2 × 2 counts); Model/covariates: age, education (if reported), otherwise “not available. Zhang (2025) [19]: AUC 0.786 (95% CI not reported); Model/covariates: “not available”. Where 95% CIs were not reported and base counts were unavailable, CIs could not be reconstructed; this is flagged in QUADAS-2/QUIPS; NR—Not Reported.

**Table 7:** Key study findings.

Study	Stroke *n* (Controls)	Demographics	Imaging Timing	Scanner and DTI Basics	Cognitive Endpoint(s) and Timing
Toh (2021) [17]	50 (44)	Age 56.7 ± 15.2 years; 60% male	MRI at 1–60 days post-onset; mean 17.1 ± 14.8 days	3 T Siemens Tim Trio; b = 1000 s/mm^2^; 20 dirs; 2 mm slices	NR
Wang (2025) [18]	29 (25)	Age 65.35 ± 11.11 years; 55.2% male	7 and 90 days after subcortical infarct	3 T Philips Ingenia; b = 1000; 32 dirs; 2 mm iso voxels	MoCA at 7 and 90 days
Zhang (2025) [19]	51 (HC 48; n-VCI 47)	NR for stroke only (overall mean age across cohorts 63 years)	Time 1 (pre-rehab) and Time 2 (≈30 days later); onset-to-scan piecewise 42-day threshold	3 T GE MR750; sequence parameters in supplement	MMSE at 6 months; PSCI at 6 months
Hong (2024) [20]	120 (—)	Age 70.0 years; 65% male	Baseline; MRI annually 3 years; cognition annually 5 years	DTI pipeline with PSMD/MD adjustment; scanner NR	Global, executive, memory, processing speed; incident dementia
Chen (2025) [21]	51 (27)	Age 53.25 ± 10.56 years; 28/51 male	3 months and 1 year	3 T Siemens Tim-Trio; controls matched; protocol detailed on PMC	Multidomain battery (language, memory, motor, attention) at both timepoints

HC—healthy controls; n-VCI—non-VCI (stroke participants without vascular cognitive impairment at assessment); DTI—diffusion tensor imaging; MoCA—Montreal Cognitive Assessment; MMSE—Mini-Mental State Examination; PSCI—post-stroke cognitive impairment; PSMD—peak width of skeletonized mean diffusivity; NR—Not Reported.

**Table 8:** Heterogeneity map of included studies (design, imaging timing, DTI parameters, ALPS ROI methods, cognitive endpoints, covariates).

Study (Year)	Design and *n* (Stroke/Controls)	Imaging Timing vs. Stroke	DTI Basics	ALPS ROI Method	Cognitive Endpoint(s) and Definition	Key Covariates/Adjustment	Notes Limiting Pooling
Toh (2021) [17]	Retrospective; 50/44	1–60 days (mean ~17 days)	3T; b = 1000; ~20 dirs; ~2 mm	Manual periventricular ROIs; side-specific	None (temporal/asymmetry only)	None for diffusion confounds	No cognitive endpoint; manual ROI; variable timing
Wang (2025) [18]	Prospective subcortical; 29/25	7 days and 90 days	3T; b = 1000; ~32 dirs; ~2 mm	Protocolized ALPS; lesion/non-lesion/bilateral	MoCA at 7 days and 90 days; early impairment per study	Limited adjustment (no PSMD/MD)	Acute/subacute only; impairment definition differs
Zhang (2025) [19]	Prospective; 51 stroke; HC 48; n-VCI 47	Time-1 pre-rehab; Time-2 ~30 days later	3T; params in supplement	Side-specific ALPS + PVS/CP metrics	6-month MMSE; PSCI per study	Partial imaging covariates	Timing windows differ; some non-numeric reporting
Hong (2024) [20]	Prospective lacunar SVD; 120	Baseline; MRI annually ×3; cognition ×5 years	Standardized DTI pipeline	Automated/standardized ALPS (global)	Global/executive/memory; incident dementia (adjudicated)	Adjusted for PSMD/MD; WMH	Outcome constructs differ; long-term SVD cohort
Chen (2025) [21]	Retrospective; 51/27	3 months and 12 months	3T; multi-direction DTI	Lesion/contralateral ROIs	Multidomain battery at 3 months and 1 years	Limited covariate adjustment	Chronic window; asymmetry resolves by 12 months

dirs—diffusion directions; HC—healthy controls; n-VCI—non-VCI stroke comparators; MoCA—Montreal Cognitive Assessment; MMSE—Mini-Mental State Examination; PSCI—post-stroke cognitive impairment; PSMD—peak width of skeletonized mean diffusivity; WMH—white-matter hyperintensity; SVD—small-vessel disease.

**Table 9:** QUADAS-2 risk-of-bias assessment for diagnostic/discriminative questions.

Study	Assessment
Toh (2021) [17]	Retrospective convenience sample; manual periventricular ROIs; blinding of ALPS readers not reported; imaging acquired across a broad 1–60 day window
Wang (2025) [18]	Prospective subcortical stroke cohort with protocolized lesion/non-lesion ROIs; blinding of ALPS measurement to MoCA not explicitly stated; fixed 7- and 90-day imaging windows; MoCA-based impairment definition used as reference standard
Zhang (2025) [19]	Prospective design but recruitment procedures and exclusions not fully detailed; side-specific ALPS with unclear ROI blinding; MMSE-based PSCI definition acceptable but briefly described; partial reporting of numeric data across timepoints
Hong (2024) [20]	Lacunar SVD cohort not designed for diagnostic classification; global ALPS derived from a standardized diffusion pipeline; dementia outcomes adjudicated; QUADAS-2 domains largely not applicable for this prognostic design
Chen (2025) [21]	Chronic-phase stroke cohort focused on longitudinal recovery; no formal diagnostic reference standard; lesion/contralateral ROIs with unclear blinding; QUADAS-2 primarily not applicable

QUADAS-2—Quality Assessment of Diagnostic Accuracy Studies 2; ALPS—analysis along the perivascular space; ROI—region of interest; MoCA—Montreal Cognitive Assessment; MMSE—Mini-Mental State Examination; PSCI—post-stroke cognitive impairment; SVD—small-vessel disease.

**Table 10:** QUIPS risk-of-bias assessment for prognostic questions.

Study	Assessment
Wang (2025) [18]	Prospective, well-defined acute subcortical cohort with short follow-up; protocolized ALPS measurement but blinding unclear; MoCA outcomes at fixed timepoints; prognostic models did not incorporate PSMD/WMH; reporting of AUCs but not all CIs
Zhang (2025) [19]	Prospective stroke cohort with incomplete reporting of recruitment and attrition; side-specific ALPS ROIs; PSCI definition based on MMSE with limited detail; imaging confounders only partially adjusted; some outcomes presented graphically without full numeric statistics
Hong (2024) [20]	Lacunar SVD cohort with standardized diffusion pipeline; automated/global ALPS; adjudicated dementia endpoints; models adjusted for PSMD/MD and WMH; multi-year follow-up with expected attrition but appropriate longitudinal analyses
Chen (2025) [21]	Retrospective chronic-stroke sample; lesion/contralateral ALPS ROIs; multidomain cognitive battery with unclear blinding; limited covariate adjustment (no PSMD/WMH); statistical models described but sparsely reported
Toh (2021) [17]	Retrospective convenience sampling; manual ALPS ROIs without reported blinding; no prognostic clinical endpoint; no adjustment for diffusion or vascular confounders; therefore high concern in multiple QUIPS domains

QUIPS—Quality In Prognostic Studies; ALPS—analysis along the perivascular space; ROI—region of interest; MoCA—Montreal Cognitive Assessment; MMSE—Mini-Mental State Examination; PSCI—post-stroke cognitive impairment; SVD—small-vessel disease; PSMD—peak width of skeletonized mean diffusivity; MD—mean diffusivity; WMH—white-matter hyperintensity; AUC—area under the receiver operating characteristic curve; CI—confidence interval.

## Data Availability

No new data were created or analyzed in this study.

## Appendix A

**Table A1:** Full electronic search strategies for each database.

Database/Platform	Search Date (Final Rerun)	Fields/Limits	Full Boolean Search String *
PubMed/MEDLINE	24 August 2025	Title/Abstract; Humans; English; Article; 1900–2025; Exclude reviews; “humans” filter active	(“DTI-ALPS”[tiab] OR “ALPS index”[tiab] OR “analysis along the perivascular space”[tiab] OR ((perivascular[tiab] OR “perivascular space”[tiab] OR “perivascular spaces”[tiab] OR “Virchow-Robin”[tiab]) AND (diffus *[tiab] OR tensor[tiab] OR DTI[tiab] OR “diffusion tensor imaging”[tiab] OR diffusivity[tiab] OR “fractional anisotropy”[tiab] OR “mean diffusivity”[tiab])) OR ((glymphatic[tiab] OR “glymphatic system”[tiab]) AND (diffus *[tiab] OR DTI[tiab] OR “diffusion tensor imaging”[tiab]))) AND (stroke[tiab] OR ischem *[tiab] OR ischaem *[tiab] OR infarct *[tiab] OR lacunar[tiab] OR “lacunar stroke”[tiab] OR “small vessel disease”[tiab] OR “small-vessel disease”[tiab] OR SVD[tiab] OR “transient ischemic attack”[tiab] OR TIA[tiab] OR lacune *[tiab] OR “subcortical infarct”[tiab] OR “subcortical infarcts”[tiab])
Scopus (Elsevier)	24 August 2025	TITLE-ABS-KEY; Document type: Article; English; 1900–2025	TITLE-ABS-KEY (“DTI-ALPS” OR “ALPS index” OR “analysis along the perivascular space” OR ((perivascular OR “perivascular space” OR “perivascular spaces” OR “Virchow-Robin”) AND (diffus * OR tensor OR DTI OR “diffusion tensor imaging” OR diffusivity OR “fractional anisotropy” OR “mean diffusivity”)) OR ((glymphatic OR “glymphatic system”) AND (diffus * OR DTI OR “diffusion tensor imaging”))) AND TITLE-ABS-KEY(stroke OR ischem * OR ischaem * OR infarct * OR lacunar OR “lacunar stroke” OR “small vessel disease” OR “small-vessel disease” OR SVD OR “transient ischemic attack” OR TIA OR lacune * OR “subcortical infarct” OR “subcortical infarcts”)
Web of Science Core Collection	24 August 2025	Topic (TS); Article; English; 1900–2025	TS = ((“DTI-ALPS” OR “DTI ALPS” OR “ALPS index” OR “ALPS-index” OR “analysis along the perivascular space” OR ((perivascular OR “perivascular space” OR “perivascular spaces” OR “Virchow-Robin”) NEAR/3 (diffus * OR tensor OR DTI OR “diffusion tensor imaging” OR diffusivity OR “fractional anisotropy” OR “mean diffusivity”)) OR ((glymphatic OR “glymphatic system”) NEAR/3 (diffus * OR DTI OR “diffusion tensor imaging”))) AND (stroke OR ischem * OR ischaem * OR infarct * OR lacunar OR “lacunar stroke” OR “small vessel disease” OR “small-vessel disease” OR SVD OR “transient ischemic attack” OR TIA OR lacune * OR “subcortical infarct” OR “subcortical infarcts”))

* TIA and small-vessel disease terms were included at the search level to maximize sensitivity; TIA-only cohorts and mixed samples without separable ischemic stroke data were excluded at full-text screening.

**Table A2:** Reasons for exclusion at full-text screening.

Reason for Exclusion at Full-Text Screening	*n*
Not ischemic stroke–specific ALPS (TIA-only or mixed cohorts without separable ischemic stroke results)	12
Wrong outcomes (no clinical, functional, cognitive, or longitudinal imaging endpoint relevant to PSCI)	8
Insufficient extractable ALPS numerical data (e.g., qualitative figures only)	4
Cohort or analysis overlap with a more complete dataset	4
Non-human or pediatric-only research	4
Total	32
